# The benefits of negative yet informative feedback

**DOI:** 10.1371/journal.pone.0205183

**Published:** 2018-10-19

**Authors:** Sung-il Kim, Suyoung Hwang, Minhye Lee

**Affiliations:** 1 Brain and Motivation Research Institute (*b*MRI), Department of Education, Korea University, Seoul, Korea; 2 Department of Teaching Education, Dankook University, Yongin, Korea; Iwate Medical University, JAPAN

## Abstract

We investigated whether negative feedback with information could benefit both behavioral and neural responses. Fifteen participants were scanned with functional magnetic resonance imaging (fMRI) while receiving various feedbacks in a novel perceptual task. Behavioral data showed that reaction times of task performance were faster after receiving negative informative feedback compared to negative confirmatory feedback. The fMRI analysis of the interaction contrast between feedback type (informative vs. confirmatory) and valence (negative vs. positive) showed greater activation in the ventrolateral prefrontal cortex (vlPFC) and the ventral striatum in response to negative informative compared to confirmatory feedback. The psychophysiological interactions (PPI) analyses showed that the vlPFC activation was positively correlated with the amygdala and the rostral cingulate zone (RCZ). The ventral striatum activation was negatively correlated with the dorsolateral prefrontal cortex (dlPFC). These results suggest that negative but informative feedback benefits subsequent performance and its primary function is to elicit positive prediction error (instructive signal) and to induce cognitive control to guide subsequent goal-directed behavior.

## Introduction

Feedback is a direct way to improve learner’s performance by providing relevant and useful information. Feedback facilitates learning and motivation because it helps the learner not only monitor performance but also adjust subsequent behavior [1]. Most psychological theories of learning and motivation posit that positive feedback generates a positive affect and fosters motivation whereas negative feedback does the opposite [2,3].

Feedback processing can be viewed from two different perspectives: reinforcement learning (focusing on positive valence of feedback) and cognitive (cybernetic) model of information processing (focusing on negative valence of feedback). According to the reinforcement learning perspective, people are strongly motivated to approach appetitive stimuli (i.e., rewards) and avoid aversive stimuli (i.e., punishment) [4]. Because positive feedback functions as an appetitive stimulus, it is likely to reinforce the target behavior by increasing the frequency with which this behavior is approached.

However, in the cognitive model, feedback serves as information used to reduce discrepancies between a target goal and current state [5]. In an attempt to learn goal-directed behavior, people need to continuously monitor ongoing performance and adopt appropriate behaviors via feedback. Thus, feedback signals, as long as they hold accurate and valuable information, are processed in the executive cortico-striatal loop to allow for behavioral adjustment. In particular, negative feedback requires executive and cognitive control processes to avoid further errors [6].

### Feedback valence

Neuroimaging studies on feedback processing have established the link between feedback valence and brain activation patterns. For example, positive feedback activates reward-sensitive areas such as the ventral striatum and the medial orbitofrontal cortex (mOFC) [7,8], while negative feedback activates the punishment-related areas of the brain, including the lateral part of the OFC [9,10], the dorsal anterior cingulate cortex (dACC) [8,11], the anterior insular cortex (AIC), and the ventrolateral prefrontal cortex (vlPFC) [12,13].

However, the relationship between feedback valence and behavior is not simple. Particularly in the case of negative feedback, several factors, including types of feedback and individual characteristics, can moderate this relationship. For example, despite the unpleasant nature of negative feedback, people often seek it out and benefit from depending on individual characteristics such as self-esteem and regulatory focus [14,15]. Furthermore, positive feedback can also undermine intrinsic motivation by increasing learner’s self-consciousness to the point of distraction from the task [16].

To understand the role of feedback in learning and motivation, many studies on feedback processing have focused on negative valence of feedback rather than positive one because the former usually informs performance adjustment and regulates behavior [6,17]. Negative feedback processing recruits the cognitive control network, a set of interconnected cortical regions such as the ACC to avoid additional errors [8,18]. A large number of studies have reported that a negative event-related potential (ERP) component, called the error-related or feedback-related negativity (ERN or FRN), occurs following negative feedback that indicates an error or incorrect response [8,19,20]. This ERN is mainly modulated by the dACC or the rostral cingulate zone (RCZ), which is known as the central brain region for attention-demanding cognitive control, including action selection and performance monitoring [21–23].

In addition, several studies have demonstrated that the mesencephalic dopamine system, including the midbrain and the ventral striatum, is often activated in response to negative feedback or aversive stimuli [24–26]. Researchers have interpreted this finding as negative feedback representing unexpected surprising information, leading to an increase in the prediction error [27]. This hypothesis has been supported by several neuroimaging studies that have demonstrated that activation of the midbrain including nucleus accumbens (NAcc) correlates with the prediction error for negative feedback [28,29].

### Feedback type

Most functional neuroimaging studies on feedback processing have focused on the differences in neural activity associated with feedback valence rather than feedback type. Although several studies have investigated the effect of types of feedback on brain activation [30], it is the informative value of feedback that determines learning and performance adjustment. Two different types of feedback can therefore be distinguished depending on the degree of information convey by the feedback: confirmatory (verification) and informative (elaborative or evaluative) feedback [31–33]. Confirmatory feedback simply indicates whether a response is correct or not. That is, it relays performance accuracy (i.e., success or failure) without any further explanation. In contrast, Informative feedback provides correction about a response by including specific reasons for the success or failure. In particular, negative informative feedback provides useful information in guiding subsequent behavior because it contains specific information about the incorrect performance that needs to be adjusted. Thus, informative feedback serves to correct errors whereas confirmatory feedback serves to confirm a correct response.

Because the success of a task performance is usually not determined by a single criterion in typical learning situations, learners may not know exactly what aspect of their performance was incorrect based solely on negative confirmatory feedback (i.e., failure). When provided informative feedback, however, learners are able to monitor their performance precisely and modify subsequent behavior [34]. Through this process, informative feedback not only helps learners engage in more detailed learning, but also it implicitly enhances their achievement motivation and task completion [35,36].

However, previous neuroimaging studies on feedback processing have mainly used confirmatory feedback by only providing the correctness of the response. Bischoff-Grethe et al. [37] found that the Ventral striatum, the caudate, the putamen, the AIC, and the supplementary motor area (SMA) were sensitive to confirmatory feedback regardless of valence. Özyurt et al. [38] also compared confirmatory feedback and no feedback and found that the rostral cingulate zone (RCZ), the vlPFC, and the insula were more strongly activated in response to confirmatory feedback than to no feedback. Although they used the term informative feedback, the feedback provided in these studies was confirmatory because they did not provide any relevant information on the participants’ performance except for indicating their successes or failures. Instead of comparing different types of feedback (confirmatory versus informative feedback), they simply compared feedback versus no feedback. Only a few studies have investigated the neural mechanisms of informative feedback processing. For example, Van den Bos et al. [39] manipulated the informative value of feedback using a probabilistic learning task. They assumed that negative feedback would contain higher informative value when participants chose the alternative rule (low probability stimuli) than the correct rule (high probability stimuli). Because negative feedback after the choice of the high probability stimuli would not provide any information for subsequent performance adjustment, it has less informative value. They found more activity in the dorsolateral prefrontal cortex (dlPFC) and the dACC (RCZ) in response to negative feedback for the alternative rule than for the correct rule, indicating that it signaled the need for cognitive control and behavior change.

Tricomi and colleagues varied the level of informational value by manipulating either the number of possible response options [6] or the predictability of the feedback reception [17]. Tricomi and Fiez [6] varied the amount of information provided by positive and negative feedback by manipulating two versus four response options. When two response options are available, positive and negative feedback provide equal amounts of information. In contrast, when four response options are available, positive feedback provides more information than negative feedback which only eliminates one of four possibilities. They found that the negative feedback in the two-response condition elicited greater signal in the caudate than four-response condition because negative feedback in the two-response condition, relative to the four-response condition, had more informational value. Lempert and Tricomi [17] also showed that negative feedback increased the caudate activity and no feedback decreased the ventral striatum activity when the receipt of feedback was unpredictable. This suggest that an unpredictable feedback context makes negative feedback more informative than predictable feedback context does.

Furthermore, Woo et al. [33] demonstrated that informative feedback serves an emotion regulation function. They presented negative feedback consisted of the image of an angry face along with the derogatory statement, ‘‘You are stupid” which induces strong negative emotion. They found that confirmatory negative feedback recruited a neural network associated with negative emotion including amygdala, whereas informative negative feedback activated the dorsolateral prefrontal cortex (dlPFC), a region involved in emotion regulation.

Although these previous studies highlighted the importance of informational value of negative feedback, it is not yet known if differential neural mechanisms underlie informative and confirmatory feedback, particularly in conjunction with a negative valence. In the present study, we directly compare informative and confirmatory feedback by varying the degree of information provided on the participants’ performance in a novel perceptual task. The difference between confirmatory and informative feedback depends on the relative amount of useful information in the feedback. The informative feedback provides more specific information which could be used to correct errors whereas confirmatory feedback provides minimal information only as to whether the response was correct or not. While informative feedback serves to correct errors, confirmatory feedback serves to confirm a correct response. Therefore, the greatest difference between confirmatory and informative feedback occurs when the feedback valence is negative.

Assuming that the ventral striatum encodes a reward prediction error signal, there would be no difference in the ventral striatal activation between positive confirmatory and positive informative feedback. Because positive feedback is a reinforcement, we hypothesized that positive feedback would produce positive reward prediction error (better-than-expected) signal regardless of feedback type, which would result in the similar activation of the ventral striatal region. However, in the case of negative feedback, the negative reward prediction error (worse-than-expected) signal would differ depending on the type of feedback. Negative confirmatory feedback would produce negative reward prediction error which would inhibit the ventral striatal activation. In contrast, it is expected that negative informative feedback would produce positive reward prediction error, resulting in greater ventral striatal activation because it contains valuable information for subsequent performance.

## Method

### Participants

Seventeen healthy, right-handed subjects participated in this fMRI experiment. None of the participants had either a history of neurological, psychiatric, or major medical disorders. The number of participants was determined by a priori power analysis with the expected strong effect size for testing difference between two dependent means using G*Power 3.1.9.2 [40]. They provided informed consent and were paid about $30 for their participation. This study was approved by the Institutional Review Board of Korea University. Two subjects were excluded from the analysis due to excessive head motion (greater than 3 mm). Thus, the final sample consisted of 15 participants (eight males, mean age = 22.21 years, SD = 1.81), which was identical with the minimum required sample size computed from the power analysis.

### Experimental stimuli and procedure

**Fig 1:**
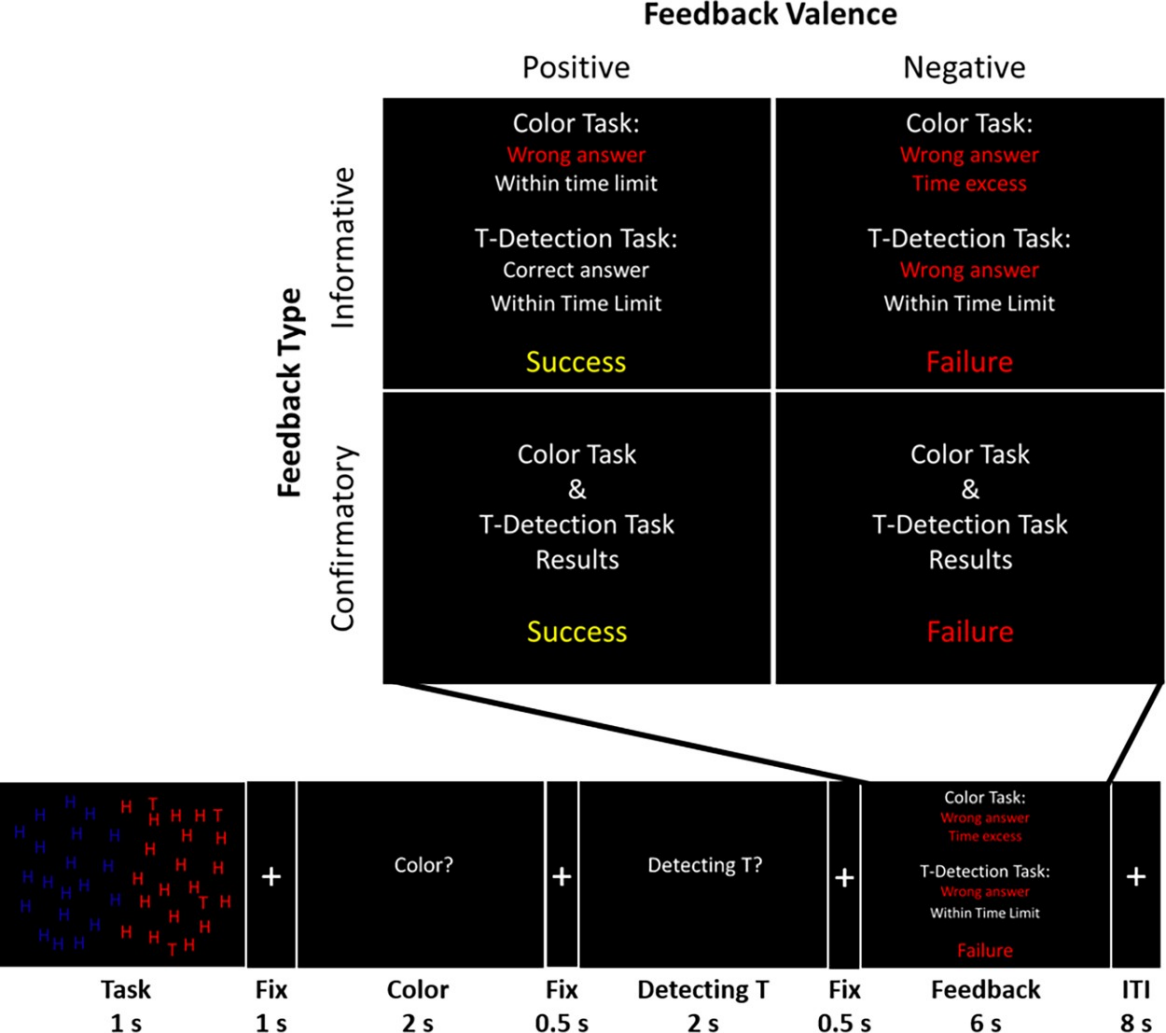
Experimental stimuli and procedure. The experimental design was a 2 (feedback type; informative vs. confirmatory) × 2 (feedback valence; positive vs. negative) event consisting of 4 runs of 20 trials each (80 trials in total). Each stimulus consisted of a large number (ranging from 25 to 30) of the letter H and a small number (ranging from 3 to 9) of the letter T which were randomly colored red or blue. Participants were presented with a stimulus of letters on the screen for 1 second and asked to perform the following two tasks consecutively: 1) a color-judgment task where they were to judge which color was more common, red or blue; and 2) a T-detection task in which they were to decide whether there were three red Ts present. After completing these tasks, participants received predetermined feedback that disregarded their actual performance to control for the amount of positive and negative feedback received. To manipulate feedback type, specific information about the criteria they met or not met was presented for informative feedback, whereas simple success or failure information was presented for confirmatory feedback. Fix = fixation; ITI = inter-trial-interval.

The experimental design was a 2 (feedback type; informative vs. confirmatory) × 2 (feedback valence; positive vs. negative) event-related design consisting of 4 runs (see Fig 1). Each run was comprised of 20 trials in which participants performed two consecutive perceptual judgment tasks and received feedback. A total of 80 trials were conducted and the scanning session took approximately 30 minutes in total. Each stimulus consisted of a large number (ranging from 25 to 30) of the letter H and a small number (ranging from 3 to 9) of the letter T, with each individual letter randomly colored red or blue (see Fig 1). Participants were presented with a stimulus of letters on the screen for 1 second and asked to perform the following two tasks consecutively: 1) a color-judgment task, where they judged which color was more numerous, red or blue; and 2) a T-detection task, where they decided whether there were three red Ts present. For the color-judgment task, participants were instructed to click the left button of the mouse if the red letters outnumbered the blue ones or click the right button if the blue letters outnumbered the red ones. For the T-detection task, if participants detected three red Ts they were to click the left button of the mouse; if not, they were to click the right one. Participants had to respond to each task within 2 seconds and the order of the two tasks was counterbalanced.

Participants were told that their response would be considered a success if the response satisfied at least three of the following four criteria: accuracy and speed for both color-judgment and T-detection tasks. To satisfy the speed criteria, participants were required to provide their response within 300 ms. Participants were informed that their response would be considered a failure if they only met one or none of the four criteria. We intentionally in excluded feedback for cases which the responses of the participants satisfied two criteria because they could not be regarded as either successes or failures.

After completing the two consecutive tasks, participants received bogus feedback unrelated to their actual performance. Unbeknownst to participants, predetermined feedback was used to control for the number of positive and negative feedback trials received by each individual. Post-experimental interviews with participants revealed that all the participants reported that they perceived the feedback as credible reflecting their actual performance. The type of feedback varied depending on the specific information presented about the performance (see Fig 1). For informative feedback, participants were told which criteria they had met and which they had not. For confirmatory feedback, participants were told whether they had succeeded or failed without any additional information. One of the four different types of feedback (positive informative, positive confirmatory, negative informative, and negative confirmatory feedback) was presented to the participants for 6 s. A random inter-trial interval varying from between 4 to 12 s (mean = 8 s) followed each feedback session.

### Imaging procedures

The functional imaging was conducted on a 3-tesla scanner (ISOL, Korea). For each subject, we acquired a whole-brain T1-weighted anatomical image (TR = 10 ms; TE = 5.7 ms, flip angle = 10°, voxel size = 1 × 1 × 1 mm^3^) and gradient echo T2*-weighted echo planar images (EPI) with BOLD contrast (TR = 2000 ms; TE = 35 ms; flip angle = 60°, sequential ascending order, FOV = 240 mm, 5-mm-thick 22-slice with no gap, 64 × 64 matrix).

### fMRI data analysis

Image analysis was performed using SPM5 (Wellcome Department of Imaging Neuroscience, Institute of Neurology, London, U.K.). To correct for head motion during scanning, images were realigned to the first image as a reference by using a least squares approach and a six-parameter fixed-body transformation. As a compromise of the 5-mm thickness of imaging option, images were then corrected for differences in the timing of slice acquisition and spatially normalized to a standard T2* template with a resampled voxel size of 2 × 2 × 2 mm^3^. Spatial smoothing was applied based on a Gaussian kernel of 8 mm full-width at half-maximum (FWHM). High-pass temporal filtering with a cut-off of 128 s was applied.

The feedback was categorized into one of four conditions (positive-informative, negative-informative, positive-confirmatory, and negative-confirmatory feedback) and modeled by convolving a delta function at each event onset with a canonical hemodynamic response function (HRF). In addition to the four feedback stimulus onset time regressors, task stimuli onset time, response times of both Color-judgment and T-detection tasks, and six head-motion parameters were included in the model.

We computed contrast images to enable comparison between positive and negative feedback and for the interaction effect between feedback type and valence at the individual level. The results from the individual analysis were taken to the second-level analyses. In general, results at a threshold of *p* < .001 uncorrected and of more than 10 voxels were considered. We also reported statistical strengths of neural responses found at a significant voxel-wise level of *p* < .05 false discovery rate (FDR) corrected for multiple comparisons. Activations in a priori region of interests (ROIs) which survived in the whole-brain correction were subject to small-volume correction (SVC). ROI masks for SVC were created based on a priori anatomical structures found in previous empirical studies. Masks for the bilateral ventral striatum and vlPFC for the interaction contrast were created based on previous studies adopting similar research designs and paradigms [12,37]. More precisely, the masks for the bilateral ventral striatum were created as 10-mm spheres centered on the coordinates (*x* = -12, *y* = 12, *z* = -8; *x* = 12, *y* = 12, *z* = -8) identified in a Bischoff-Grethe et al.’s empirical study [37]. The mask for the bilateral vlPFC was also created as a 10-mm sphere centered on the coordinates (*x* = 32, *y* = 24, *z* = 2; *x* = -34, *y* = 22, *z* = 0) reported in a Monchi et al.’s study [12]. These regions are closely associated with the interaction effects between feedback valence and type. We also conducted ROI analyses by using the MarsBar SPM Toolbox (http://www.sourceforge.net/projects/marsbar) to infer and visualize the activation patterns of the regions identified in the target interaction contrasts. In order to confirm the independence between whole-brain results and functional ROIs, we utilized the leave-one-subject-out (LOSO) method [41]. To explore functional connectivity with the ROIs, we also conducted psychophysiological interactions (PPI) analyses for the target interaction contrast, (negative informative–positive informative) > (negative confirmatory–positive confirmatory). MNI coordinates were converted into the standard space of Talairach and Tournoux [42] when we reported the anatomical locations of significant activation foci.

## Results

### Behavioral results

**Fig 2:**
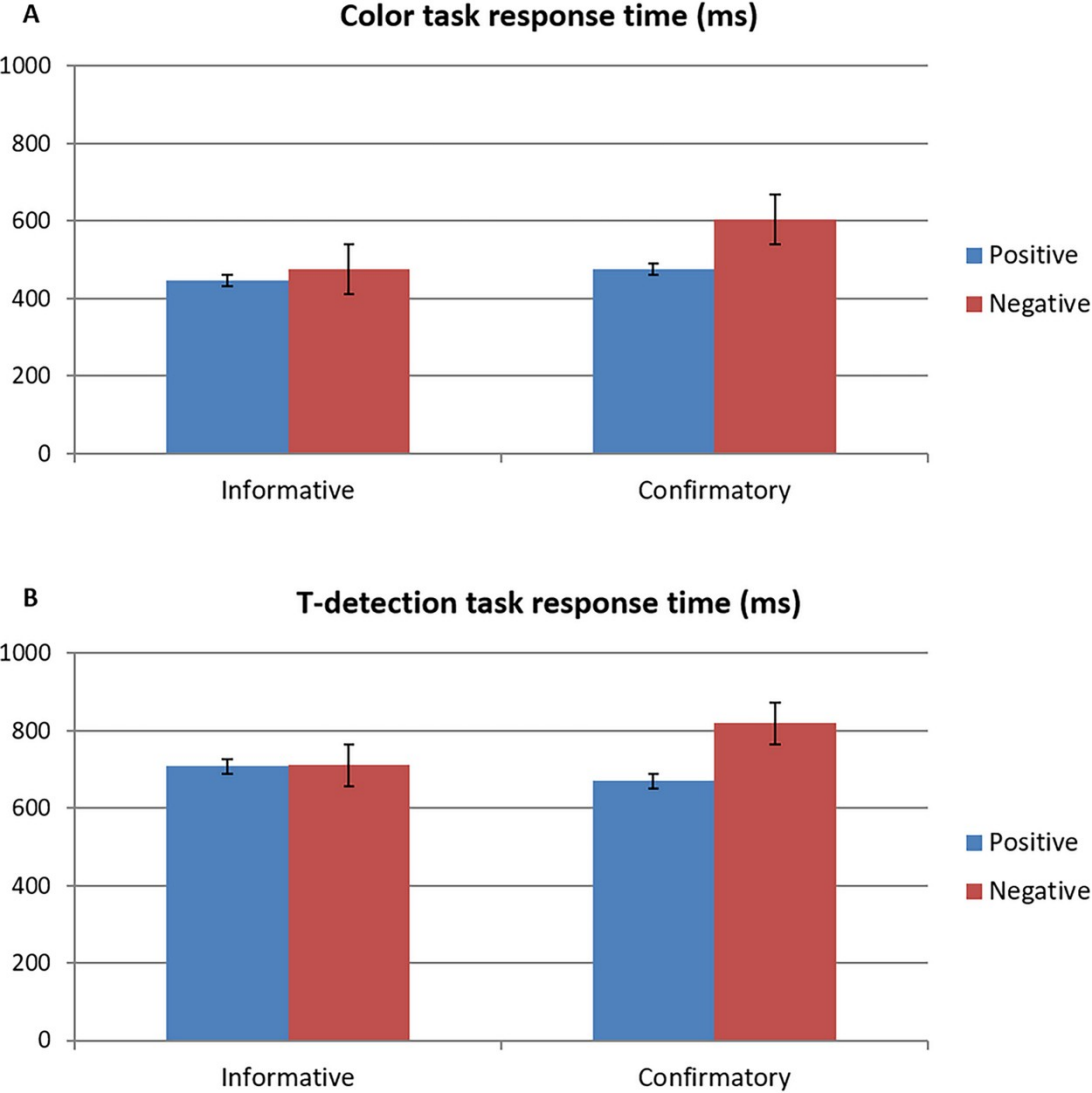
Interaction effect of feedback type and valence on response time of (A) color-judgment task and (B) T-detection task.

We conducted repeated-measure ANOVAs on reaction time and accuracy of color-judgment task and T-detection task, respectively (see Table 1 and Fig 2A and 2B). To examine the effect of prior feedback on subsequent task performance, we analyzed reaction time and accuracy for task trials immediately following the feedback. There were significant interaction effects between feedback type and valence on response time of both tasks (for color task, *F*_(1, 14)_ = 78.92, *p* < .01, ŋ^2^ = .85; for T-task, *F*_(1, 14)_ = 24.66, *p* < .01, ŋ^2^ = .64), but not on task accuracy (for color task, *F*_(1, 14)_ = .00, *p* = .99, ŋ^2^ = .00; for T-task, *F*_(1, 14)_ = .51, *p* = .49, ŋ^2^ = .04). For confirmatory feedback, negative feedback produced a longer reaction time than did positive feedback (for color task, *t*_(14)_ = 11.01, *p* < .01; for T-task, *t*_(14)_ = 7.07, *p* < .01); however, for informative feedback, no difference was found between positive and negative feedback in both tasks. In addition, we found main effects of feedback valence on response time of both tasks (for color task, *F*_(1, 14)_ = 47.07, *p* < .01, ŋ^2^ = .77; for T-task, *F*_(1, 14)_ = 30.61, *p* < .01, ŋ^2^ = .69, respectively) and main effects of feedback type on the color task accuracy (*F*_(1, 14)_ = 5.09, *p* < .05, ŋ^2^ = .27) and on the T-detection task reaction time (*F*_(1, 14)_ = 22.11, *p* < .01, ŋ^2^ = .61).

**Table 1:** Descriptive statistics for reaction time and accuracy for each task.

Feedback type	Feedback valence	Reaction time		Accuracy	
		*Mean*	*SD*	*Mean*	*SD*
***Color-judgment task***					
Informative	Positive	708.55	206.18	51.50	15.30
	Negative	711.09	190.66	52.38	5.97
Confirmatory	Positive	670.13	161.17	46.62	11.05
	Negative	819.02	175.65	47.50	6.48
***T-detection task***					
Informative	Positive	447.28	128.39	53.01	9.64
	Negative	475.34	149.72	55.44	14.01
Confirmatory	Positive	476.79	177.04	53.38	13.90
	Negative	604.40	182.14	51.07	11.68

### fMRI results

#### Whole brain analyses

**Fig 3:**
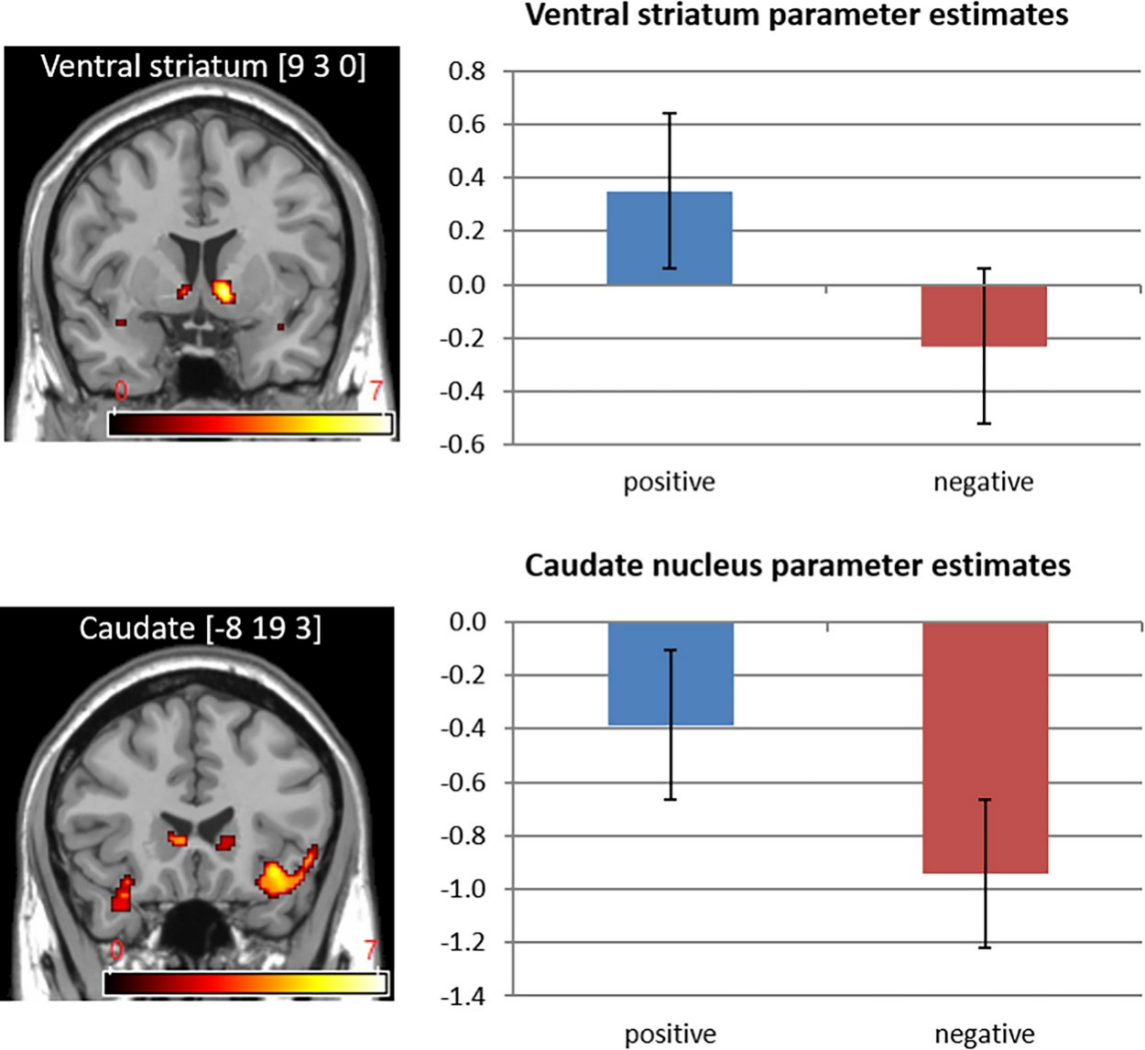
Striatum activations in the (Positive > Negative) contrast.

We examined neural differences based on feedback valence and type (see Tables 2 and 3, respectively). In the (positive feedback > negative feedback) contrast, individuals showed increased activation in the ventral striatum, caudate nucleus, cerebellum, anterior cingulate cortex, superior temporal gyrus, and parahippocampal gyrus (*p* < .001 uncorrected, with a minimum cluster size of 10 voxels) when receiving positive feedback compared to receiving negative feedback (see Fig 3). On the contrary, for the (negative feedback > positive feedback) contrast, participants showed greater activation only in the posterior insula when receiving negative feedback than when receiving positive feedback. In regard with the contrast of (informative feedback > confirmatory feedback), participants had greater activations in the ACC, inferior parietal lobule, caudate nucleus, AIC, and cerebellum when receiving informative feedback compared to receiving confirmatory feedback. In contrast, regarding the brain activations in the (confirmatory feedback > informative feedback) contrast, participants showed greater activation in the cerebellum, superior temporal gyrus, and cuneus when receiving confirmatory feedback than when receiving informative feedback.

**Table 2:** Feedback valence contrasts.

Region	R/L	BA	Talairach Coordinate			Voxel (*k*)	*z-*value
			*x*	*y*	*z*		
***Positive > Negative feedback***							
Ventral striatum	R	48	9	3	0	79	4.56
	L	48	-8	0	1	32	3.44
Cerebellum	R		23	-41	-11	81	4.07
Inferior temporal gyrus	R	19	47	-72	-1	43	3.85
Superior temporal gyrus	L	38	-34	12	-22	194	3.83
Parahippocampal gyrus	R	34	11	-8	-13	56	3.76
Anterior cingulate cortex	R	9	3	35	31	54	3.63
	R	40	58	-31	40	18	3.44
Cuneus	R	18	12	-98	16	16	3.62
Inferior parietal lobe	L	40	-54	-38	41	20	3.57
Caudate nucleus	L	48	-8	19	3	54	3.54
	R	48	13	15	4	22	3.48
Thalamus	R	50	1	-15	-1	25	3.51
Medial globus pallidus	R		15	-8	1	21	3.46
Inferior frontal gyrus	R	46	46	36	8	16	3.44
Middle frontal gyrus	L	10	-39	38	0	11	3.36
Supramarginal gyrus	R	40	56	-45	35	37	3.35
Posterior cingulate cortex	R	23	8	-50	21	22	3.33
***Negative > Positive feedback***							
Posterior insula	L	13	-43	-17	5	58	3.57
Medial frontal gyrus	R	6	8	-3	59	18	3.47
	L	6	-1	-8	61	15	3.43

*p* < .001 uncorrected, extent threshold *k* > 10.

**Table 3:** Feedback type contrasts.

Region	R/L	BA	Talairach Coordinate			Voxel (*k*)	*z-*value
			*x*	*y*	*z*		
***Informative > Confirmatory feedback***							
Anterior cingulate cortex*	R	6	12	14	46	8540	5.95
Inferior parietal lobule*	R	40	55	-29	38	3103	4.78
Superior temporal gyrus*	L	22	-60	-47	15	28	4.29
Caudate nucleus*	L	48	-7	0	11	222	4.25
Cerebellum*	L		-27	-70	-12	143	3.65
Anterior insula*	L	13	-33	18	-5	80	3.62
Inferior occipital gyrus*	L	19	-43	-74	-5	25	3.44
Middle temporal gyrus*	L	37	-51	-51	-7	16	3.32
Thalamus*	R	50	11	-13	3	13	3.22
***Confirmatory > Informative feedback***							
Cerebellum	L		-13	-61	-4	753	4.46
Superior temporal gyrus	R	22	45	-21	6	188	3.76
Cuneus	R	31	2	-66	12	130	3.49
Lingual gyrus	L	18	-14	-75	7	15	3.36
Posterior cingulate cortex	L	23	-7	-48	26	20	3.21

* *p* < .05 FDR corrected.

*p* < .001 uncorrected, extent threshold *k* > 10.

To investigate the interaction effect between feedback type and valence, we created contrasts between negative and positive feedback within each type of feedback for the second-level analysis (see Table 4). In the contrast of (negative informative feedback–positive informative feedback) > (negative confirmatory feedback–positive confirmatory feedback), brain activations in the left vlPFC and the right ventral striatum reflect the interaction effect between feedback type and valence (*p* < .001 with a minimum cluster size of 10 voxels; *p* < .05 FDR small volume corrected). In the contrast of (negative confirmatory–positive confirmatory) > (negative informative–positive informative), brain activations in the left posterior insular cortex, left calcarine sulcus, and right middle temporal gyrus also reflect the interaction effects between feedback type and valence (*p* < .001 with a minimum cluster size of 10 voxels).

**Table 4:** Interaction effect of feedback type and valence.

Region	R/L	BA	Talairach Coordinate			Voxel (*k*)	*z-*value
			*x*	*y*	*z*		
**(*NeInfo—PoInfo) > (NeConf—PoConf)***							
Ventrolateral prefrontal cortex*	L	47	-31	25	4	25	3.72
Ventral striatum*	R	52	11	6	-9	20	3.61
Hippocampus	R	54	29	-16	-9	17	3.57
***(NeConf—PoConf) > (NeInfo—PoInfo)***							
Posterior insula	L	13	-40	-19	8	167	4.47
Calcarine sulcus	L	17	-22	-73	14	32	4.02
Fusiform gyrus	L	19	-31	-69	1	13	3.89
Middle temporal gyrus	R	21	49	-6	-2	21	3.35
Precuneus	R	7	6	-58	53	16	3.43

*Note*. NeInfo = negative informative feedback; PoInfo = positive informative feedback; NeConf = negative confirmatory feedback; PoConf = positive confirmatory feedback.

* *p* < .05 FDR small volume corrected.

*p* < .001 uncorrected, extent threshold of *k* > 10.

#### Functional ROI analyses

**Fig 4:**
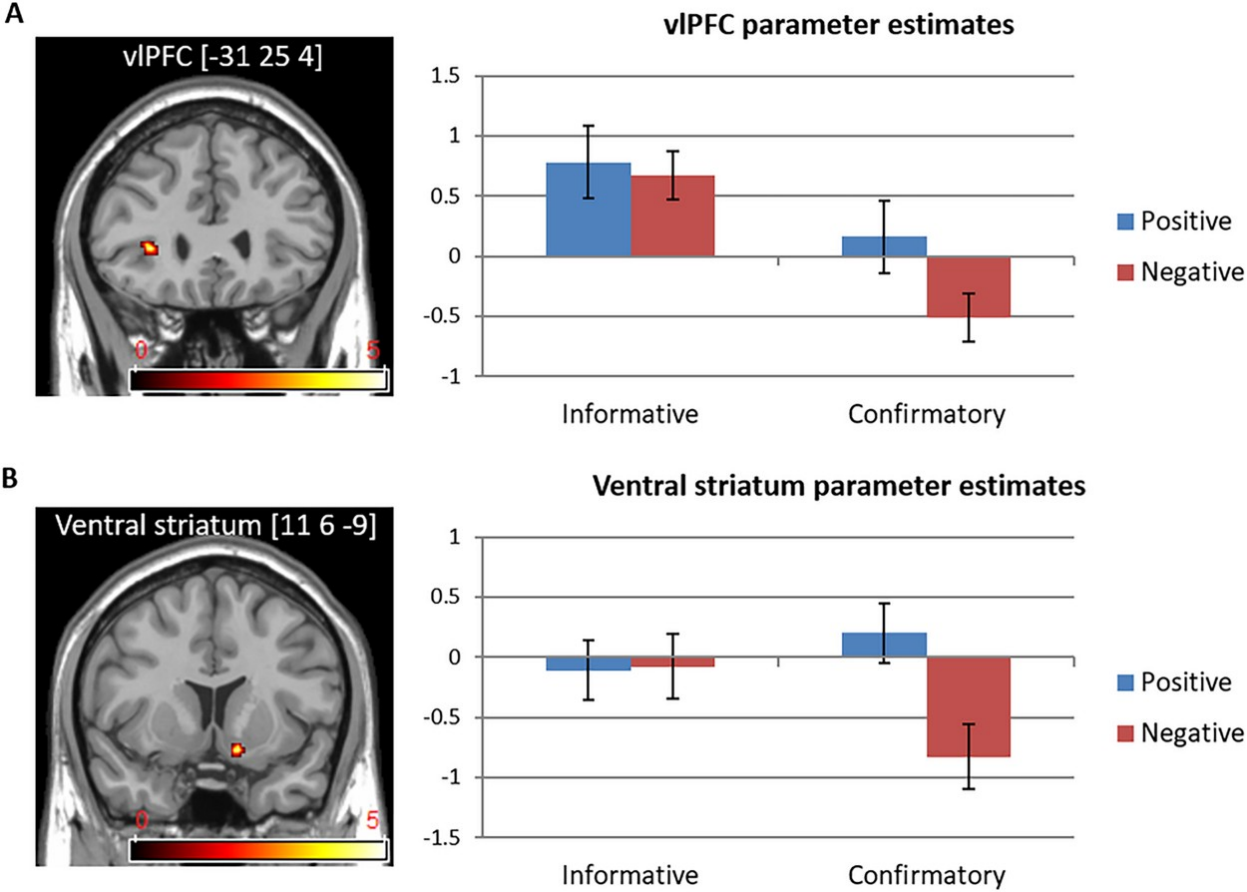
(A) Left vlPFC and (B) right ventral striatum activations in the (Negative Informative–Positive Informative) > (Negative Confirmatory–Positive Confirmatory) contrast.

We performed functional ROI analyses on those regions found to be significant in the interaction analysis by using the LOSO method. We found that the brain activation patterns in the ROI varied depending on feedback valence in the case of confirmatory feedback, whereas this was not the case for informative feedback. For confirmatory feedback particularly, positive feedback showed increased activity in the left vlPFC (*F*_(1, 14)_ = 13.38, *p* < .05, ŋ^2^ = .49; see Fig 4A) and the right ventral striatum (*F*_(1, 14)_ = 17.21, *p* < .01, ŋ^2^ = .55; see Fig 4B), compared to negative feedback. However, the valence of informative feedback did not affect the brain activation patterns in the ROI. In contrast, the left posterior insular cortex showed heightened activation when participants received negative confirmatory feedback (*F*_(1, 14)_ = 4.79, *p* < .05, ŋ^2^ = .26). The left calcarine sulcus showed decreased activation when participants received negative informative feedback, whereas the other types of feedback did not lead to the meaningful changes in the calcarine sulcus activation (*F*_(1, 14)_ = 5.62, *p* < .05, ŋ^2^ = .29).

#### PPI analyses

To explore the brain connectivity with the ROIs from the (negative informative–positive informative) > (negative confirmatory–positive confirmatory) contrast, we conducted PPI analyses with the left vlPFC and right ventral striatum as the seed regions (see Table 5). PPI analyses showed that the vlPFC activation was positively correlated with the right amygdala, and the right rostral cingulate zone (RCZ) (*p* < .001 uncorrected, *k* > 10). The ventral striatum activation was negatively correlated with the right dorsolateral prefrontal cortex (dlPFC) (*p* < .001 uncorrected, *k* > 10).

**Table 5:** Psychophysiological interactions with the vlPFC and ventral striatum activations as the seed ROIs from (Negative Informative–Positive Informative) > (Negative Confirmatory–Positive Confirmatory) contrast.

Region	R/L	BA	Talairach Coordinate			Voxel (*k*)	*z-*value
			*x*	*y*	*z*		
***Positive correlation with the left vlPFC***							
Amygdala	R	53	32	-1	-13	65	3.86
Rostral cingulate zone	R	32	13	21	25	99	3.68
Dorsolateral prefrontal cortex	L	9	-21	35	22	13	3.59
	R	10	27	41	17	16	3.33
***Negative correlation with the right ventral striatum***							
Dorsolateral prefrontal cortex	R	9	28	45	37	129	4.54
Posterior cingulate cortex	L	23	-1	-42	32	16	3.38

*p* < .001 uncorrected, extent threshold of *k* > 10.

## Discussion

The main objective of the present study was to investigate whether negative feedback with information can benefit learning and performance. We compared both behavioral and neural responses to different types and valences of feedback while performing a novel perceptual task. Behavioral data showed that reaction times of task performance were faster after receiving positive confirmatory feedback compared to confirmatory feedback. However, for informative feedback, no significant differences in reaction time were found between negative and positive feedback, indicating that negative informative feedback serves the same function as positive informative feedback. The interaction between feedback valence and type in reaction time suggests that negative feedback containing information about learners’ current performance can benefit subsequent performance, whereas negative confirmatory negative feedback without specific information does not.

The fMRI interaction contrast between feedback type (informative-confirmatory) and valence (negative-positive) revealed significant activation in the vlPFC and the ventral striatum. ROI analyses revealed that confirmatory feedback elicited lower activation in the vlPFC and the ventral striatum during negative feedback than it did during positive feedback, whereas informative feedback showed the same activation pattern in these regions regardless of valence.

Consistent with the well-established link between the ventral striatum and positive feedback [43], we found greater ventral striatum activity in response to positive feedback compared to negative feedback. However, there was no difference in the ventral striatum activation between positive and negative informative feedback. Because negative informative feedback contains unexpected but relevant information for subsequent performance, it would be rewarding and produce positive reward prediction error (better-than-expected) signal in the ventral striatum. In contrast, negative confirmatory feedback would produce negative reward prediction error (worse-than-expected) signal. Since the ventral striatum has anticipatory or evaluative function [44] and plays a critical role in integrating appetitive and aversive predictions [27,29], the current finding may reflect the prediction error signal in the ventral striatum during feedback-based learning [45]. This is in line with the recent argument that the ventral striatum encodes action outcomes that guide the direction of subsequent ones by integrating cognitive and affective information especially in ambiguous and uncertain situations [46]. This suggests that the ventral striatum activity increases as long as the feedback provides appropriate instructive signals for learning regardless of the valence of the feedback.

In addition, the PPI analysis revealed that the ventral striatum activation during negative informative feedback was negatively correlated to the activation of dlPFC. This negative functional connectivity between the ventral striatum and the dlPFC seems to be inconsistent with the previous findings demonstrating dopaminergic modulation over dlPFC [47]. One possibility is that the dlPFC activation, a region involved in working memory might be decreased when the ventral striatum was relatively more activated due to positive prediction error signal. Negative informative feedback might enhance performance (faster reaction time and higher accuracy) by inhibiting redundant working memory function.

We also found an interaction effect between feedback type and valence in vlPFC activation. In this study, vlPFC activation was found in response to informative feedback regardless of valence. For confirmatory feedback, however, the vlPFC had relatively lower activation in response to negative feedback than to positive feedback. This is inconsistent with previous findings of greater vlPFC activation following negative feedback compared to positive feedback [10,48]. In the current study negative confirmatory feedback did not provide any specific information that helped the adjustment of subsequent performance. For example, when participants received negative confirmatory feedback, they did not know which criteria they failed to satisfy. In contrast, positive confirmatory feedback indicated whether they had met most of the criteria (3 or 4 criteria). Therefore, in this study, negative confirmatory feedback had less informative value than positive confirmatory feedback. This suggests that the vlPFC is sensitive to the informative value of feedback rather than the valence and that the vlPFC is recruited only when feedback carries significant information for future goal-directed behavior.

Although the lateral PFC has been implicated in cognitive control, recent evidence suggests functional dissociation within the subregions of the lateral PFC [49,50]. Monchi et al. [12] distinguished the role of the vlPFC from that of the dlPFC and vmPFC. They specified that during reversal learning tasks the vlPFC is recruited when planning a response to negative feedback, whereas the vmPFC is activated in response to emotionally salient feedback. They also stated that the dlPFC is involved in the monitoring of information held in working memory, whereas the vlPFC is recruited during the active comparison of stimuli held in working memory. Petrides [51] argued that the vlPFC is involved in the active maintenance of relevant information, whereas the dlPFC is modulated by more complex higher order information processing. Badre and colleagues found that the left vlPFC was implicated in the top-down control of goal-relevant knowledge [52,53].

Although the RCZ, known as a performance-monitoring region [23], was not significantly activated in response to negative informative feedback, the subsequent PPI analysis revealed that the vlPFC activation during negative informative feedback was positively correlated to the activation of the RCZ. The indirect involvement of the RCZ indicates that negative informative feedback might enhance top-down cognitive control including performance-monitoring.

Interestingly, the amygdala activation was also found to be positively correlated to the vlPFC activation. This positive amygdala-vlPFC functional connectivity suggests that the vlPFC might involve in the regulation of negative affect elicited by negative informative feedback. This is congruent with current claims that functional coupling between the amygdala and the vlPFC was associated with decreased anxiety [54,55]. Taken together, the PPI results suggest that negative informative feedback enhances both performance monitoring and goal maintenance via emotion regulation such as anxiety-buffering. Although further studies are needed to delineate the precise function of the vlPFC during negative informative feedback processing, we speculate that the main function of the vlPFC might be an active maintenance of task-relevant feedback information.

In summary, negative feedback is beneficial for improving subsequent performance when it conveys relevant information. The greater activation of the vlPFC and the ventral striatum in response to informative negative feedback in comparison to confirmatory negative feedback suggests that the primary function of negative informative feedback is to elicit positive prediction error (instructive signals) and to induce cognitive control to guide subsequent goal-directed behavior.

Our findings have important implications for instructional and educational practices. Negative feedback could be beneficial for learning and motivation in any learning context as long as it is provided with relevant information for future performance. Furthermore, it would be worthwhile to investigate further how individual characteristics of the learner interact with various types of feedback. The potential limitations of the present study stem from small sample size. Although our sample could have been stronger, it is in the marginally acceptable range to provide valid and reliable knowledge about interactions between feedback type and valence. Another limitation is that we used bogus feedback regardless of participants’ actual performance. Although all the participants believed that the feedback was based on their actual performance, it is necessary to conduct future research using different learning task with real feedback.

## Supporting information

S1 File Whole-brain results from the [Informative > Confirmatory] contrast. (IMG)

S2 File Whole-brain results from the [Confirmatory > Informative] contrast. (IMG)

S3 File Whole-brain results from the [Positive > Negative] contrast. (IMG)

S4 File Whole-brain results from the [Negative > Positive] contrast. (IMG)

S5 File Whole-brain results from the [(Negative Informative–Positive Informative) > (Negative Confirmatory–Positive Confirmatory)] contrast. (IMG)

S6 File Whole-brain results from the [(Negative Confirmatory–Positive Confirmatory) > (Negative Informative–Positive Informative)] contrast. (IMG)
